# Computational design of a multiepitope vaccine targeting VP1 and VP2 capsid proteins of simian virus 40 (SV40) for enhanced immune activation

**DOI:** 10.1515/med-2025-1284

**Published:** 2026-03-18

**Authors:** Muhammad Naveed, Muhammad Asim, Tariq Aziz, Sonia Amjad, Muhammad Nouman Majeed, Syed Babar Jamal, Rania Ali El Hadi Mohamed, Maher S. Alwethaynani, Fakhria A. Al-Joufi, Deema Fallatah, Shaza N. Alkhatib

**Affiliations:** Department of Biotechnology, University of Central Punjab, Faculty of Science and Technology, Lahore, Punjab, Pakistan; Laboratory of Animal Health Hygiene and Food Quality University of Ioannina Arta, 47132, Arta, Greece; Department of Biological Sciences, National University of Medical Sciences Rawalpindi Pakistan, Rawalpindi, Pakistan; Department of Biology, College of Science, Princess Nourah bint Abdulrahman University, P.O Box 84428, Riyadh, 11671, Saudi Arabia; Department of Clinical Laboratory Sciences, College of Applied Medical Sciences, Shaqra University, Alquwayiyah, Riyadh, Saudi Arabia; Department of Pharmacology, College of Pharmacy, Jouf University, Aljouf, Saudi Arabia; Department of Medical Laboratory Sciences, College of Applied Medical Sciences, Prince Sattam Bin Abdulaziz University, Al-Kharj, Saudi Arabia; Department of Biological Sciences, College of Sciences and Arts Khulais, University of Jeddah, Jeddah, Saudi Arabia

**Keywords:** simian virus, multi-epitope vaccine, TLR3, molecular docking, molecular dynamics simulation

## Abstract

**Objectives:**

This study aimed to design and evaluate a computationally constructed multiepitope vaccine targeting Simian Virus 40 (SV40) by predicting and selecting immunogenic B-cell and T-cell epitopes derived from the VP1 and VP2 capsid proteins using bioinformatics approaches.

**Methods:**

B and T-cell epitopes from VP1 and VP2 were predicted and screened for antigenicity, non-allergenicity, and non-toxicity. Structural modeling and validation were performed using PSIPRED, trRosetta, and a Ramachandran plot. Population coverage was assessed using the IEDB. Molecular docking with TLR3 and TLR5, immune simulations, *in silico* cloning, and molecular dynamics simulations were used to evaluate binding, expression, and structural stability.

**Results:**

Molecular docking with human receptors TLR3 and TLR5, revealing strong binding affinities of −1,008.3 kcal/mol and −1,309.2, and further validated using MD simulation analysis. The *in silico* expression analysis, performed using the SnapGene tool, indicated high expression levels in the pBR322 vector. The immune simulation analysis showed that the vaccine has a high capacity to induce an immune response in the host.

**Conclusions:**

The designed vaccine demonstrated high immunogenic potential; further *in vitro* and *in vivo* studies are needed to verify the antigenic potential and safety of the designed vaccine.

## Introduction

Simian Virus 40 (SV40) is a polyomavirus first identified in 1960 and later detected in polio vaccines produced using monkey kidney cells, resulting in the exposure of approximately 100 million individuals between 1955 and 1963 [1]. SV40 belongs to the *Polyomaviridae *family, has an icosahedral morphology, and possesses a double-stranded DNA genome that encodes seven proteins, including the large T antigen, which is crucial for viral replication and oncogenic processes [2].

Studies have shown a higher correlation between SV40 and malignancies, such as mesothelioma and non-Hodgkin lymphoma, in the United States and Italy. In contrast, Turkey demonstrates little to no association, likely due to the delayed adoption of polio vaccines. In 2020, four cases were reported in the USA and two cases in Italy [3]. An estimated 10–30% of polio vaccine lots in the USA were reported to contain SV40 [4].

Mostly, SV40 infections exhibit no symptoms; however, they can sometimes cause respiratory infections, fever, and flu-like symptoms in individuals with compromised immune systems [5]. The historical exposure to SV40 and the possibility of its enduring existence in human tissues are still being investigated, even though SV40 has been removed from the production of vaccines. SV40 encodes the large T antigen, a highly oncogenic protein that can disable crucial tumor suppressors, such as p53 and retinoblastoma (Rb), resulting in excessive cell division and cancer [6]. Epidemiological studies have shown a linkage between SV40 and cancers, including brain tumors, mesotheliomas, bone tumors, and non-Hodgkin lymphoma, especially in areas where contaminated vaccines were widely used [1]. The link between SV40 and human tumors continues to be debated with controversy. Some researchers argue that the finding of SV40 DNA within tumor tissues is the result of contamination at various levels of laboratory processes or through procedures related to the polymerase chain reaction (PCR), rather than infection by the virus. Regardless, this possibility underscores the importance of ongoing molecular monitoring of SV40 due to its oncogenic properties, even if the virus is not currently widely disseminated [3].

The prevalence of SV40 in human specimens ranges from 1.3 % to 25.6 %, with detection in urine, blood, and tissue specimens [7]. SV40 was first transmitted to humans through contaminated vaccines; however, modern studies suggest that it can also spread through feces, respiration, and maternal-child interaction [8]. The transmission of SV40 from Human to Human has yet to be studied. However, its DNA has been found in urine, blood, and tissue samples, with some studies suggesting the possibility of low-level persistence or reactivation [9]. There is currently no licensed vaccine or specific antiviral treatment available for SV40 infection, and the long-term effects of historical exposure in individuals remained undetermined [10]. Considering the oncogenic potential of SV40, along with the lack of preventive measures, there is a need to design a vaccine that would serve both immunotherapeutic and prophylactic purposes for elderly cancer patients and other immunosuppressed individuals who were historically exposed to radiation.

Currently, no SV40-specific antiviral drugs or vaccines are available against SV40. However, several therapeutic strategies are being explored, particularly in the context of gene therapy and cancer treatment [11]. The small molecular inhibitors, such as MAL2-11B, have shown potential in targeting the ATPase activity of the SV40 large T antigen. Fluoroquinolones have some ability to inhibit the helicase function of the SV40 T-antigen, though the integration risks of recombinant SV40 vectors in gene therapy are a consideration [12]. Researchers are exploring the combination of conventional cancer treatments with innovative approaches, such as virotherapy and immunotherapy, with a particular interest in using oncolytic viruses alongside standard therapies to improve outcomes in SV40-associated tumors [13]. However, the most promising solution is to reduce SV40-associated malignancies by enhancing the adaptive immune response through the development of a preventive vaccine, thereby preventing the persistent and tumorigenic effects of SV40.

The development of a multiepitope vaccine is essential to overcome the risks associated with SV40, mainly its association with severe tumors that can be fatal. Recent advances in computational vaccinology have revolutionized traditional vaccine development by enabling rapid, cost-effective, and precise identification of vaccine antigens [14]. *In silico* methods enable the prediction of B-cell and T-cell epitopes, facilitate structural modeling, and support immune profiling, as well as perform immune profiling and vaccine optimization with greater accuracy, eliminating the need for immediate laboratory resources [15]. These methods also lower the risk of developing potential allergens and toxic compounds, and significantly accelerate the preclinical development phase [16]. Multiepitope vaccines represent an advancing area of investigation, as vaccines engineered through *in silico* analysis may evoke cellular immune responses and yield substantial immunological efficacy [17]. The incorporation of T-cell epitopes derived from viral proteins could address the antigenic complexity in vaccines. An effective vaccine must provoke both T cells and B cells that can enhance the immune responses aimed at eradicating viral antigens present within the host organism [18]. This approach is particularly advantageous for SV40, a virus for which no vaccine currently exists, and experimental research remains limited due to its oncogenic risk.

This study aims to develop a computationally based multiepitope vaccine targeting the VP1 and VP2 capsid proteins of SV40, as done in previous studies by Enayatkhani et al. (2021) against COVID-19 [19], multiepitope vaccine against Nipah virus by Naveed et al. (2023) [20], and in multiepitope vaccine study against Monkeypox virus by Nayak et al. (2024) [21]. By targeting the VP1 and VP2 capsid proteins and integrating antigenicity, allergenicity, and structural validation tools, this work introduces a systematic pipeline for vaccine development against a historically neglected but potentially oncogenic virus. The innovation lies in combining molecular docking, immune simulation, and molecular dynamics to validate the vaccine’s immunogenicity and stability, thereby providing a foundational step toward future experimental validation.

## Materials and methods

**Figure 1:**
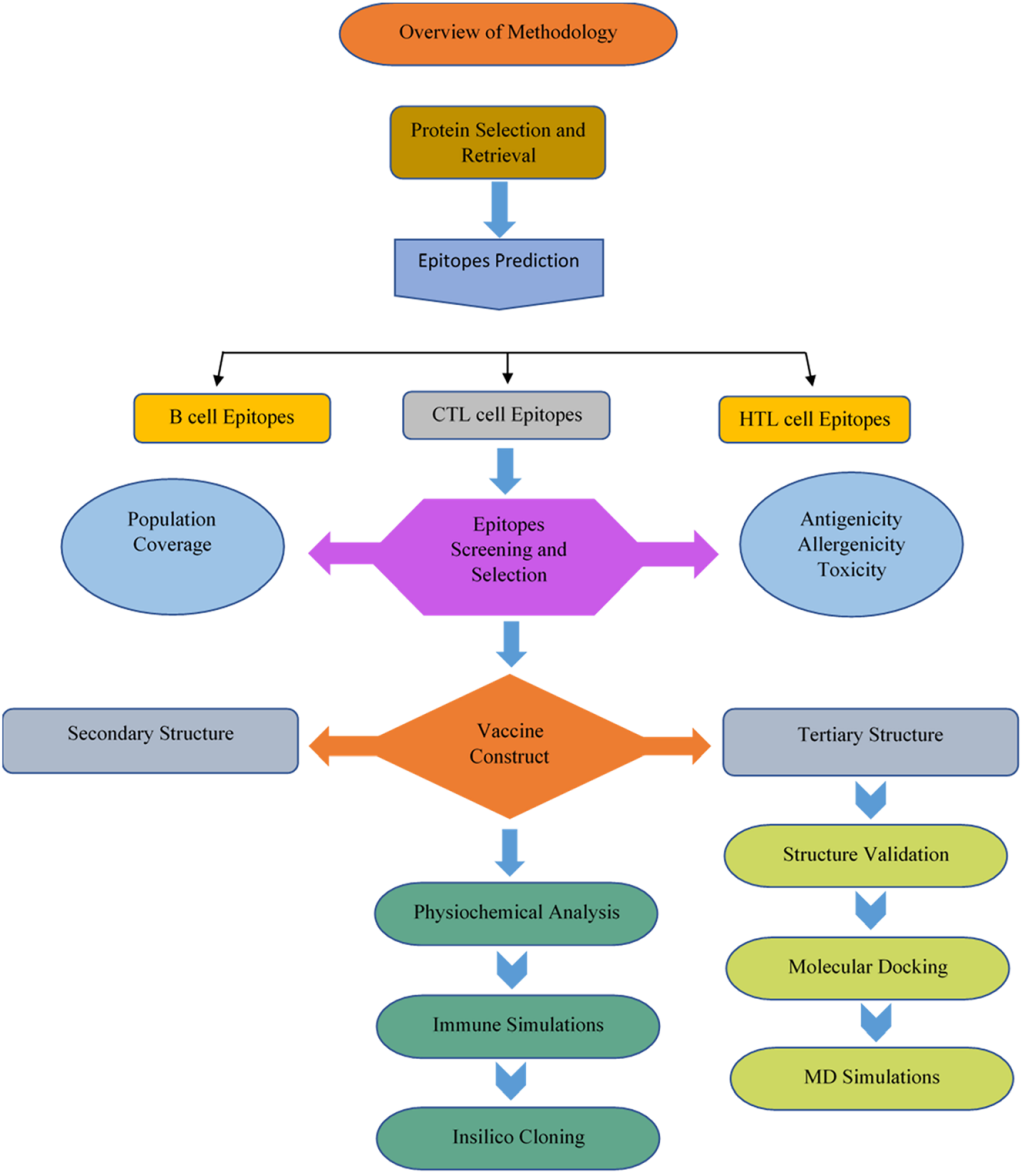
Flow chart representing the overview of methodology.

The procedure for designing a multiepitope vaccine for SV40 began with the retrieval of SV40 protein sequences for VP1 and VP2. These sequences were analyzed for B-cell and T-cell epitope prediction and screening based on antigenicity, allergenicity, and toxicity. The chosen epitopes were combined to form a multiepitope vaccine, incorporating an adjuvant and linkers to enhance structural stability and immunogenicity. The vaccine construct was evaluated for immunological parameters, including antigenicity, allergenicity, and population coverage. Structural modeling was performed at both the secondary and tertiary levels, followed by structural refinement and validation. Immune simulations were then performed to predict host immune responses, and *in silico* codon optimization and cloning were carried out to ensure expression compatibility in *Escherichia coli* K12. The molecular dynamics simulations evaluated the structural equilibrium and overall stability of the vaccine-receptor complex. This integrative *in silico* pipeline enabled the systematic and rational design of a promising multiepitope vaccine candidate targeting SV40. The overall methodology used in this study is summarized in Figure 1.

### Retrieval of targeted proteins

The initial step in developing a multiepitope vaccine candidate is the retrieval of target proteins. The FASTA sequences of Simian Virus 40 proteins were retrieved from the UniProt database (https://www.uniprot.org/) [22].

### Prediction of B-cell epitopes

B-cell epitopes are essential because they are involved in the production of antibodies that specifically target antigens. The prediction of B-cell epitopes for the selected transmembrane proteins was performed utilizing the Bepipred Linear Epitope Prediction 2.0 tool (http://tools.iedb.org/bcell/). Potential linear B-cell epitopes were selected using a threshold of 0.5 to maximize the specificity and reliability of the selection process [23].

### Prediction of T-cell epitopes

To predict epitopes of MHC class 1 (CTL), the IEDB server (http://tools.iedb.org/main/tcell/) was utilized using the ANN 4.0 methodology. This method identified numerous epitopes, effectively distinguishing between epitopes and non-epitopes, and yielded precise predictions. Furthermore, the analysis incorporated IC50 < 100 nM cutoff values for strong binders. For the prediction of MHC class II epitopes, the NN-align 2.3 (Net MHCII 2.3) method from the IEDB server (http://tools.iedb.org/mhcii/) was used. The epitopes were sorted based on IC50 <100 nM. The epitopes were further screened based on predictions of allergenicity, antigenicity, and toxicity. The prediction of these characteristics ensures the safety of the selected epitopes [24].

### Multiepitope vaccine assemblage

The identified B-cell epitopes, MHC class I, and class II epitopes were combined using linkers to form the vaccine construct. The GPGPG, AAY, and KK linkers were used to link epitopes, and these linkers are known to reduce steric hindrance and facilitate proper protein folding, ensuring effective immune recognition [25]. Beta-defensin-3 (NCBI accession: NP_061131.1) was used as an adjuvant to improve antigen uptake by antigen-presenting cells [26]. Moreover, a PADRE sequence was added to the C-terminal end of the vaccine construct to enhance immune response, and a 6× histidine tag was added at the C-terminus to facilitate the detection and purification of the vaccine construct in an expression system [27].

### Population coverage analysis

The IEDB Population Coverage Tool (https://www.iedb.org/) was used to determine the population coverage of MHC class I and II epitopes. This analysis determined the binding strength of chosen epitopes to major histocompatibility complex (MHC) alleles within specific populations. The aim was to discover epitopes that are most capable of ensuring adequate demographic coverage with an appropriate immune response among various populations [28].

### Immunocompatibility of vaccine

For antigenic evaluation of the vaccine, the VaxiJen v2.0 web server (http://www.ddgpharmfac.net/vaxijen/VaxiJen/VaxiJen.html) was used, with a threshold of 0.5, to classify probable antigens from non-antigens. The AllerTOP v2.1 server (https://www.ddg-pharmfac.net/AllerTOP/) was used to predict the allergenic potential of the vaccine. It utilizes auto cross-covariance (ACC) transformation, which estimates an allergen risk by converting peptide sequences into vector representations [29]. The CSM-Toxin Tool was used to predict the toxicity of the vaccine construct. The vaccine sequence was provided as input to the CSM-Toxin web server (https://biosig.lab.uq.edu.au/csm_toxin/), which utilizes machine learning models for toxicity predictions that incorporate both structural and physicochemical properties of a substance. This tool analyzed the sequence and computed a toxicity score using the default settings, which predicted whether the protein is toxic or non-toxic.

### Physicochemical properties analysis

The physicochemical properties of the vaccine construct were analyzed using the ProtParam tool (https://web.expasy.org/protparam/). The analyzed parameters included the construct’s molecular weight, aliphatic index, solubility, and half-life. Other essential parameters, such as the grand average of hydropathy (GRAVY) and theoretical isoelectric point (pI), were calculated to assess the vaccine’s functional and structural stability [30].

### Prediction of solubility

The solubility of the vaccine construct was estimated with the help of the SoluProt v1.0 tool available at (https://loschmidt.chemi.muni.cz/soluprot/) with a threshold of 0.5. This computational tool predicts protein solubility based on its amino acid sequence, determining whether a specific protein is likely to be soluble or insoluble under particular conditions [31], 32].

### Prediction of secondary structure

The PSIPRED server was used to predict the secondary structure of the vaccine construct (http://bioinf.cs.ucl.ac.uk/psipred/). This server utilizes position-specific scoring matrices in conjunction with PSI-BLAST to identify and classify the structural components of protein sequences, thereby predicting their two-dimensional structure [33]. The GOR IV (https://npsa-pbil.ibcp.fr/cgi-bin/npsa_automat.pl?page=/NPSA/npsa_gor4.html) server was used to cross-validate the secondary structure. This method predicts three fundamental structures: α-helices, β-sheets, and coils by performing a detailed statistical analysis of nearby amino acid sequences [34].

### Prediction of tertiary structure

The trRosetta server (https://yanglab.qd.sdu.edu.cn/trRosetta/) was employed for 3D vaccine structure prediction. It predicts the spatial relationships of amino acid residues in proteins. By utilizing these predicted inter-residue distances and orientations, trRosetta generates highly accurate 3D protein structure models [35].

### Refinement and validations of structure

The predicted tertiary structure of the vaccine was refined by the GalaxyRefine2 web server (http://galaxy.seoklab.org/cgi-bin/submit.cgi?type=REFINE). This tool enhances structural accuracy by optimizing atomic interactions and minimizing steric clashes [28]. The refined 3D model was validated using the PROCHECK server (http://services.mbi.ucla.edu/SAVES/), which computes the stereochemical integrity of the 3D model through Ramachandran plot analysis [36].

### Molecular docking and interaction analysis

Using ClusPro 2.0 (https://cluspro.org/tut_dock.php), molecular docking was performed to investigate the interaction between human toll-like receptor 3 (TLR3), toll-like receptor 5 (TLR5), and the designed vaccine construct [37]. ClusPro 2.0 utilizes energy-based algorithms to predict optimal binding positions, considering the involved protein-protein interaction energies and geometrical complementarity [38]. The docking complex was visualized using Discovery Studio v24.1.0, whereas LigPlot+v2.2.4 was used to visualize and analyze the interactions of vaccine-TLR complex, including hydrogen bonding and hydrophobic interactions, providing insights into the stability and binding efficiency of the vaccine-receptor interaction [39].

### Immune simulations

The immune-stimulating capability of the designed vaccine construct was conducted using the C-ImmSim server (https://kraken.iac.rm.cnr.it/C-IMMSIM/index.php) with three different doses. This program utilizes agent-based modeling and machine learning to simulate immune interactions and predict vaccine-mediated adaptive immune responses. Specific parameters, such as a random seed value of 12345, the simulation volume of 10, and 1,050 simulation steps, were defined for the simulation. Three injections of the vaccine (excluding lipopolysaccharide, LPS) were simulated at time steps 1, 84, and 180, representing primary and booster doses. Each injection was provided with 1,000 adjuvant units of the vaccine construct and at a concentration of 100 units to enable synergistic immune response activation [40].

### Codon optimization and *In silico* cloning

The EMBOSS Backtranseq tool (https://www.ebi.ac.uk/jdispatcher/st/emboss_backtranseq) was used to reverse-translate the vaccine sequence into its corresponding nucleotide sequence. To enhance the expression efficiency, codon optimization was performed using the codon optimization (ExpOptimizer) (https://www.novoprolabs.com/tools/codon-optimization), ensuring compatibility with the host system *E.coli* K12 [41]. *In silico* cloning of the optimized vaccine sequence was done using SnapGene v8.0.0 software. The vaccine construct was cloned into the pBR322 plasmid vector, which aids in the expression and stability of the vaccine construct [42].

### Molecular dynamics simulations

The AMBER suite of computational programs was used for molecular dynamics (MD) simulations to investigate the dynamic behavior of the docked complex between the vaccine and the TLR3 receptor over 100 ns. The docking complex was solvated using a TIP3P water model within a simulation box extended by 12 Å in all three directions from the solute. Sodium and chloride ions were strategically incorporated to achieve system neutrality and physiological conditions [43]. Following an extensive 20,000-step energy minimization to resolve any steric clashes or unfavorable interactions, the system undergoes a vital equilibration phase lasting 5 ns at a constant temperature of 298 K and pressure of 1 bar, utilizing an integration timestep of 2 fs. These precise pressure and temperature conditions were maintained throughout the production MD simulations, enabling the collection of 2,000 frames of trajectory data at intervals of 10 ps. Different parameters, including Root Mean Square Deviation (RMSD), Root Mean Square Fluctuation (RMSF), Radius of Gyration (Rg), Principal Component Analysis (PCA), and Hydrogen Bonding (H-bonding), were calculated from the simulation analysis [44].

### Ethical approval/clinical trial number

Ethical approval or Clinical Trial number does not apply to this study as it does not include the participation of humans or animals.

## Results

### Retrieval of targeted proteins

The two capsid proteins of SV40, including major capsid protein VP1 with UniProt ID P03087 and Minor capsid protein VP2 with UniProt ID P03093, were selected and obtained in FASTA format from the UniProt database for vaccine construct design.

### Prediction of B-cell epitopes

For the VP1 and VP2 proteins, the IEDB server predicted numerous B-cell epitopes. B-cell epitopes were screened out based on their higher antigenic ability and non-allergenicity. A single B-cell epitope was selected for inclusion in the vaccine design from each protein. The details of selected epitopes are presented in Table 1. The shortlisted epitopes scored 0.7568 and 1.03, which are above the threshold of 0.5, indicating strong antigenic potential. In addition, both epitopes were confirmed to be non-allergenic and non-toxic.

**Table 1:** Screened and selected B cell epitopes.

Protein	Epitope	Antigenic score	Allergenicity	Toxicity
VP1	TSGTQQWKGLP	**0.7568**	Non-allergen	Non-toxic
VP2	FSDWDHKVSTVGIYQQP	1.0351	Non-allergen	Non-toxic

### Prediction of T-cell epitopes

The predicted epitopes of MHC class I were shortlisted based on IC50 values. Following this, epitopes were chosen based on their heightened antigenicity scores, which ranged from 0.5 to 1.08, alongside criteria of non-allergenicity and toxicity. For MHC-II epitope prediction, the IEDB server was configured to utilize the default parameters, with an epitope length set at 15. The resulting epitopes were subsequently arranged according to their adjusted ranking. The epitopes were then screened and selected based on their antigenic score, allergenicity, and toxicity profile. Table 2 and Table 3 represent the selected epitopes of MHC-I and-II, which exhibit elevated antigenicity scores, non-allergenicity, and non-toxicity.

**Table 2:** Selected epitopes of MHC class I.

Protein	Epitope	Alleles	Antigenicity	Allergenicity	Toxicity
VP1	VLDKDNAYPV	HLA-A^a^02:01, HLA-A^a^02:06, HLA-A^a^02:03	0.9984	Non-allergen	Non-toxic
	IQGSNFHFF	HLA-B^a^15:01, HLA-A^a^23:01, HLA-A^a^24:02	0.7638	Non-allergen	Non-toxic
VP2	SEAIAAIGL	HLA-B^a^40:01, HLA-B^a^44:02	0.8384	Non-allergen	Non-toxic
	KTKGTSASAK	HLA-A^a^30:01, HLA-A^a^03:01	1.0849	Non-allergen	Non-toxic

**Table 3:** Selected epitopes of MHC class II.

Protein	Epitope	Alleles	Antigenicity	Allergenicity	Toxicity
VP1	FHFFAVGGEPLELQG	HLA-DRB1^a^01:01, HLA-DRB1^a^07:01, HLA-DRB1^a^09:01	0.9686	Non-allergen	Non-toxin
	SNFHFFAVGGEPLEL	HLA-DRB1^a^01:01, HLA-DRB1^a^07:01, HLA-DRB1^a^09:01, HLA-DRB1^a^04:05	1.2303	Non-allergen	Non-toxin
VP2	AIAAIGLIPQAYAVI	HLA-DRB1^a^01:01, HLA-DRB1^a^12:01, HLA-DRB1^a^08:02, HLA-DRB4^a^01:01	0.8929	Non-allergen	Non-toxin
	QDYYSTLSPIRPTMV	HLA-DRB1^a^01:01, HLA-DRB5^a^01:01, HLA-DRB1^a^07:01, HLA-DRB1^a^09:01, HLA-DRB1^a^04:01, HLA-DRB1^a^04:05	0.9405	Non-allergen	Non-toxin

### Multiepitope vaccine assemblage

**Figure 2:**
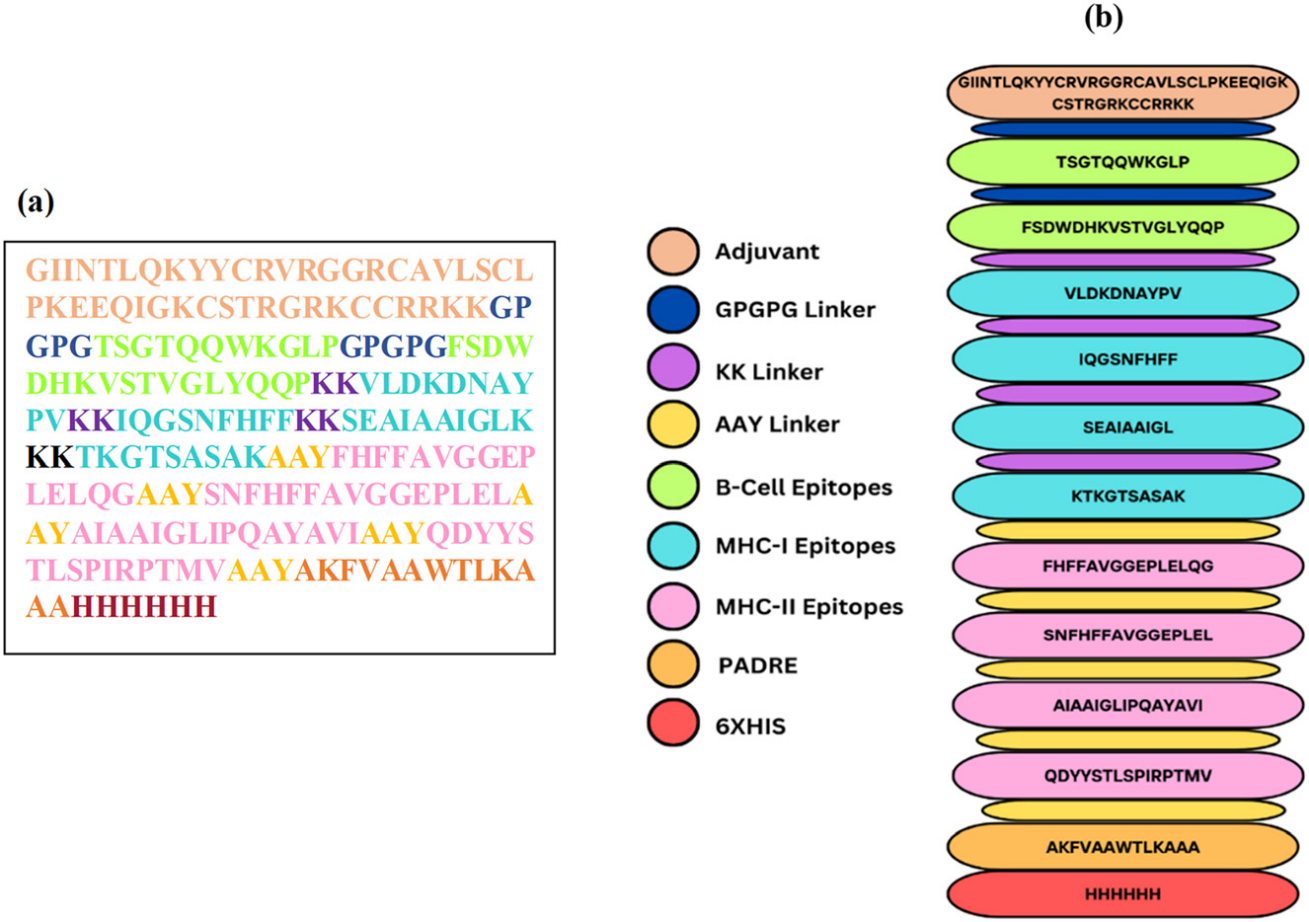
Designed vaccine construct: (a) Linear amino acid sequence of designed vaccine containing 198 amino acids. (b) Schematic diagram of designed vaccine construct.

The vaccine construct, shown in Figure 2, consists of an adjuvant sequence, linkers including GPGPG, KK, and AAY, which were used to join the B-cell and T-cell epitopes, a PADRE sequence, and a 6 × His tag. The construction of the vaccine sequence began with the β-defensin sequence as an adjuvant. B-cell epitopes were linked utilizing a GPGPG linker, while MHC class I and MHC class II epitopes were joined through KK and AAY linkers. A 6 × His tag is integrated at the C-terminus to facilitate purification and protein identification. The final vaccine sequence comprised a total of 223 amino acids.

### Immune compatibility of vaccine

The antigenicity of the designed vaccine candidate was assessed using the VaxiJen v2.0 server, which yielded an antigenicity score of 0.7120. This score indicates that the vaccine has the potential to elicit an immune response within the host. Furthermore, allergenicity analysis using AllerTOP v2.1 confirmed that the vaccine is non-allergenic. Additionally, toxicity evaluation using the CSM-Toxin tool determined that the vaccine is non-toxic and considered safe for human use.

### Population coverage analysis

**Figure 3:**
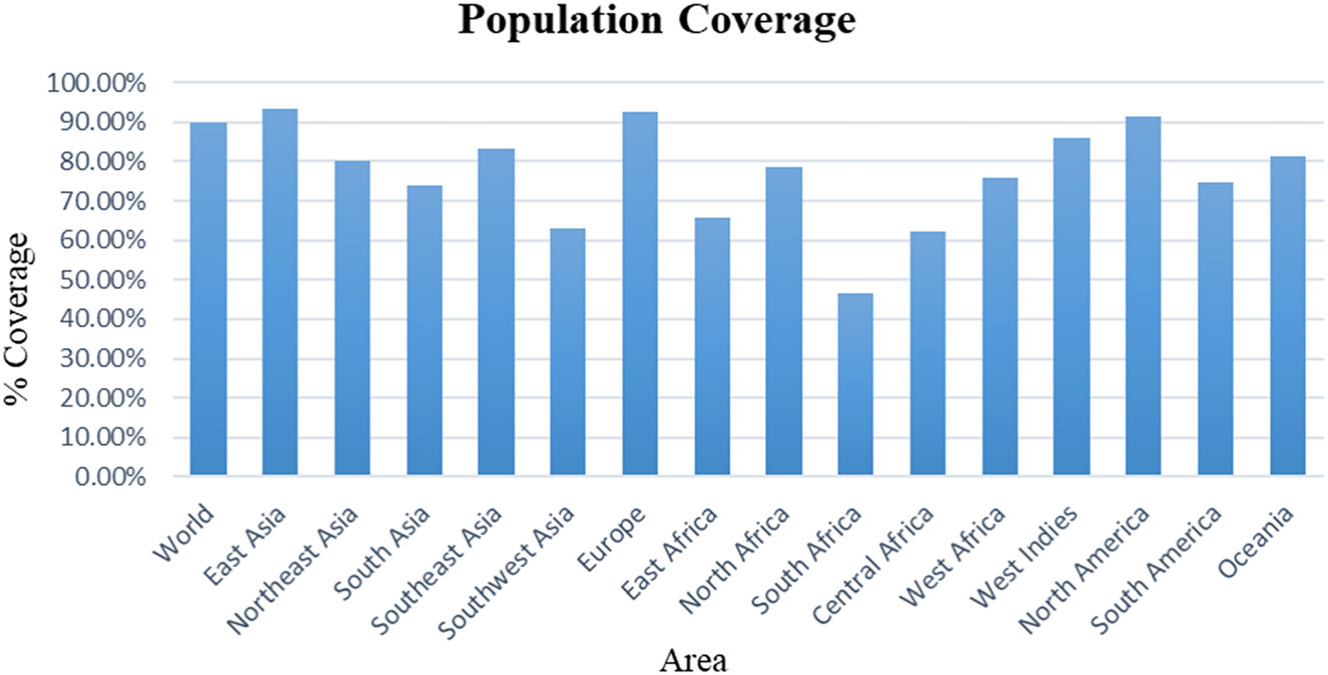
Population coverage analysis of T-cell epitopes across different regions of world.

The cumulative population coverage of MHC class I and MHC class II class epitopes, shown in Figure 3, demonstrates a high global coverage of 89.07 %, indicating its global immunogenic potential across diverse populations. The vaccine showed high coverage in East Asia (94.7 %), including countries like China, Japan, and South Korea. Populations from Europe and North America showed 91.2 % coverage, while Latin America (including Mexico, Brazil, and Argentina) had 89.0 %. South Asia, comprising India, Pakistan, and Bangladesh, showed 83.5 % coverage. In contrast, Central Africa exhibited lower coverage at 68.4 %, likely due to greater HLA diversity. These results indicate broad global coverage of the vaccine, though the inclusion of additional epitopes may further improve responses in African populations.

### Physicochemical properties analysis

As predicted by the ExPASy ProtParam, the physicochemical characteristics of the vaccine candidate have a molecular mass of 24,243.96 Da, comprising 223 amino acid residues and a total of 3,415 atoms, with a theoretical isoelectric point (pI) of 9.73. The ProtParam analysis indicated that the protein exhibits stability, as evidenced by an instability index of 26.26. The Grand Average of Hydropathicity (GRAVY) value was calculated to be −0.265, indicating the hydrophilic characteristics of the protein. At the same time, the aliphatic index was appraised at 71.48, suggesting a degree of thermostability for the protein. The vaccine half-life in *Escherichia coli* (*in vivo*) was determined to be greater than 10 h. Furthermore, the vaccine demonstrated a high level of solubility in *Escherichia coli*, achieving a solubility score of 0.876, as assessed by the SoluProt tool. The physicochemical attributes of the vaccine protein are illustrated in Table 4.

**Table 4:** Physiochemical properties of vaccine protein.

Physiochemical properties	
**Number of amino acids**	223
Molecular weight	24243.96Da
Theoretical pI	9.73
Total number of negatively charged residues (Asp+Glu)	12
Total number of positively charged residues (Arg+Lys)	30
Total number of atoms	3,415
Aliphatic index	71.48
Grand average of hydropathicity (GRAVY)	−0.265
Instability index	26.26
Estimated half life in *E.coli* (*in vivo*)	>10 h
Solubility	0.876

### Secondary structure prediction

**Figure 4:**
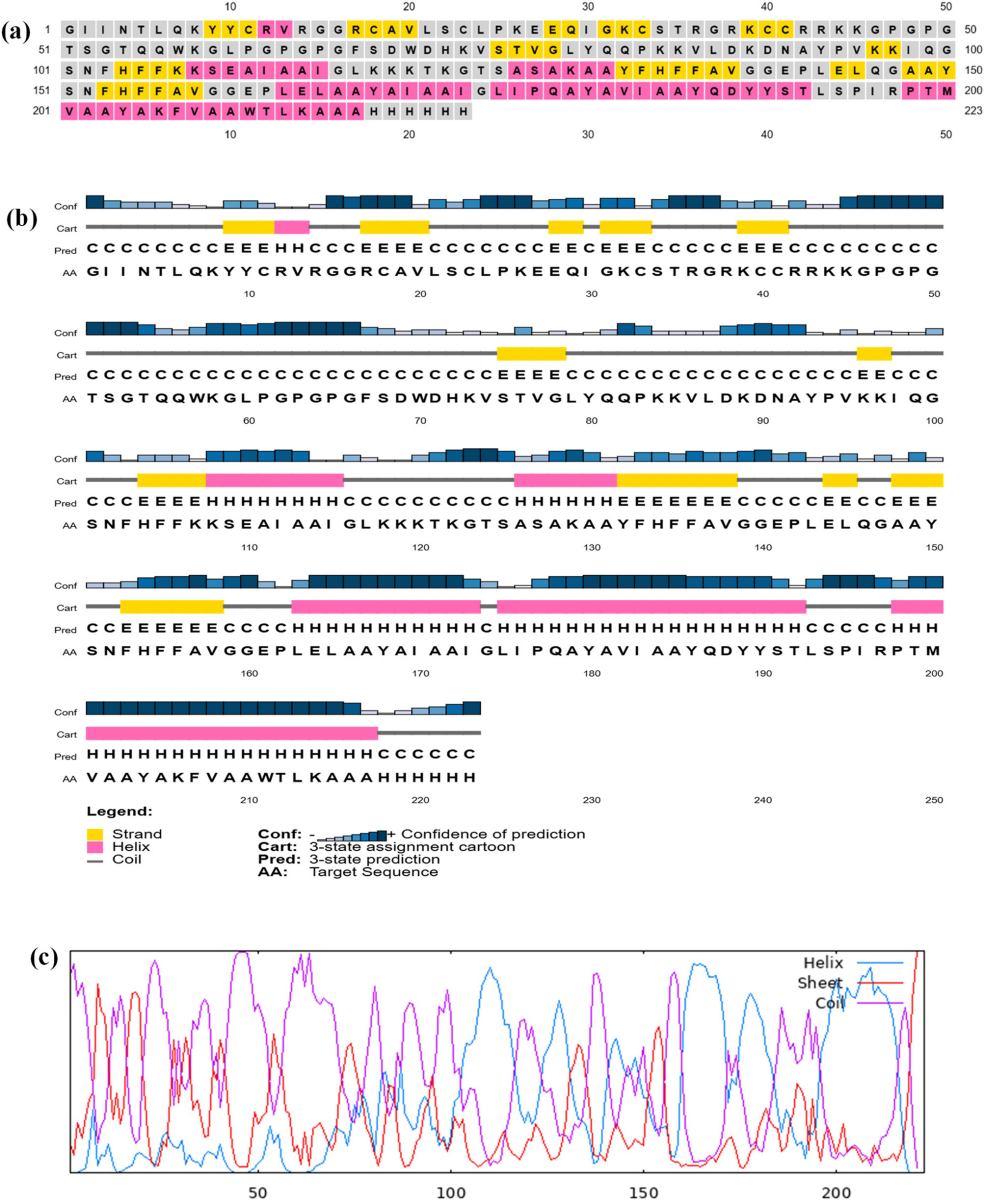
Secondary structure prediction of the multiepitope vaccine construct. (a) Secondary structure of vaccine construct predicted by PSIPRED server. (b) PSIPRED cartoon image of vaccine sequence. (c) GORIV secondary structure prediction, red lines representing alpha helix and blue lines displaying beta strand and pink lines indicating coils.

The 2D structure of the vaccine, as predicted by PSIPRED, reveals the presence of helices (depicted in pink), coils (illustrated in grey), and sheets (represented in yellow), with a significant proportion of the protein’s amino acid composition corresponding to coils, alpha helices, and a smaller fraction contributing to the formation of beta sheets, as demonstrated in Figure 4a. The overall proportion of α-helices is calculated to be 38.49 %; extended strands constitute 13.45 %. In comparison, random coils account for 56.05 % of the secondary structure as interpreted through the GOR IV tool, as shown in Figure 4b and c.

### Tertiary structure prediction and validation

**Figure 5:**
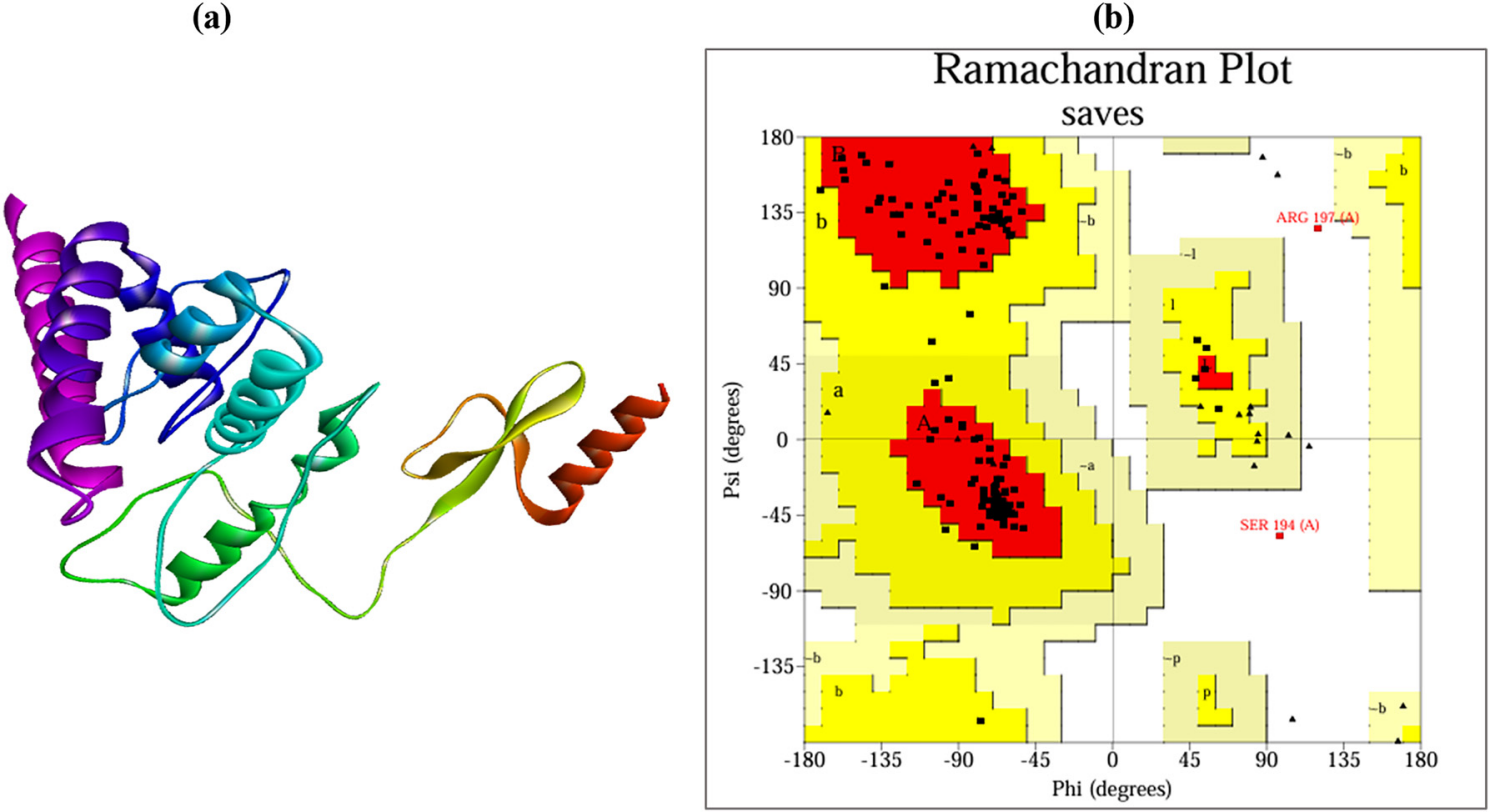
Tertiary structure prediction and validation of the vaccine construct. (a) Tertiary structure of vaccine predicted by trRosetta and refined by GalaxyRefine2. (b) Ramachandran plot showing 94.6 % of residues in most favored regions.

The 3D structure obtained from trRosetta with a TM-score of 0.464 is shown in Figure 5a. The predicted model underwent refinement through the GalaxyRefine2 server. After refinement, the Rama favor score improved from 94.4 % to 96.8 %. The initial model, characterized by an RMSD score of 0.326, was obtained from the GalaxyRefine2 server. The model was subjected to further validation using a ramachandran plot, which demonstrated that 91.9 % of the amino acid residues were located in the most favorable region, 7 % in the allowed region, and 1.1 % in the disallowed region, as shown in Figure 5b.

### Molecular docking and interaction analysis

**Figure 6:**
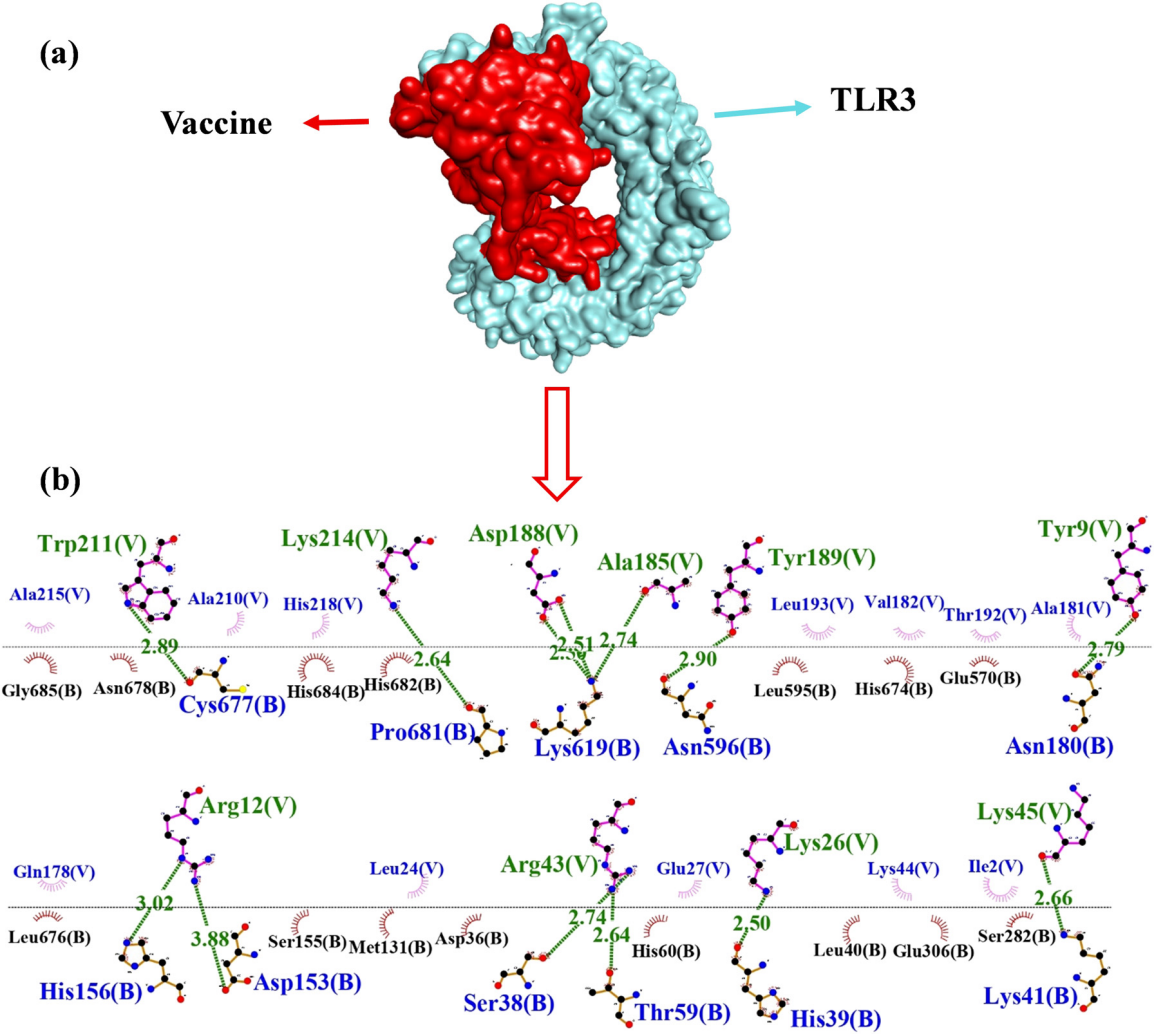
Molecular interaction between the vaccine construct and TLR3 receptor. (a) Docking complex of vaccine candidate with TLR3 receptor visualized by discovery studio. (b) Docking interaction analyzed through LigPlot+v2.2.4 showing the hydrogen bonds with green lines between amino acid residues of TLR-3 receptor and vaccine.

**Figure 7:**
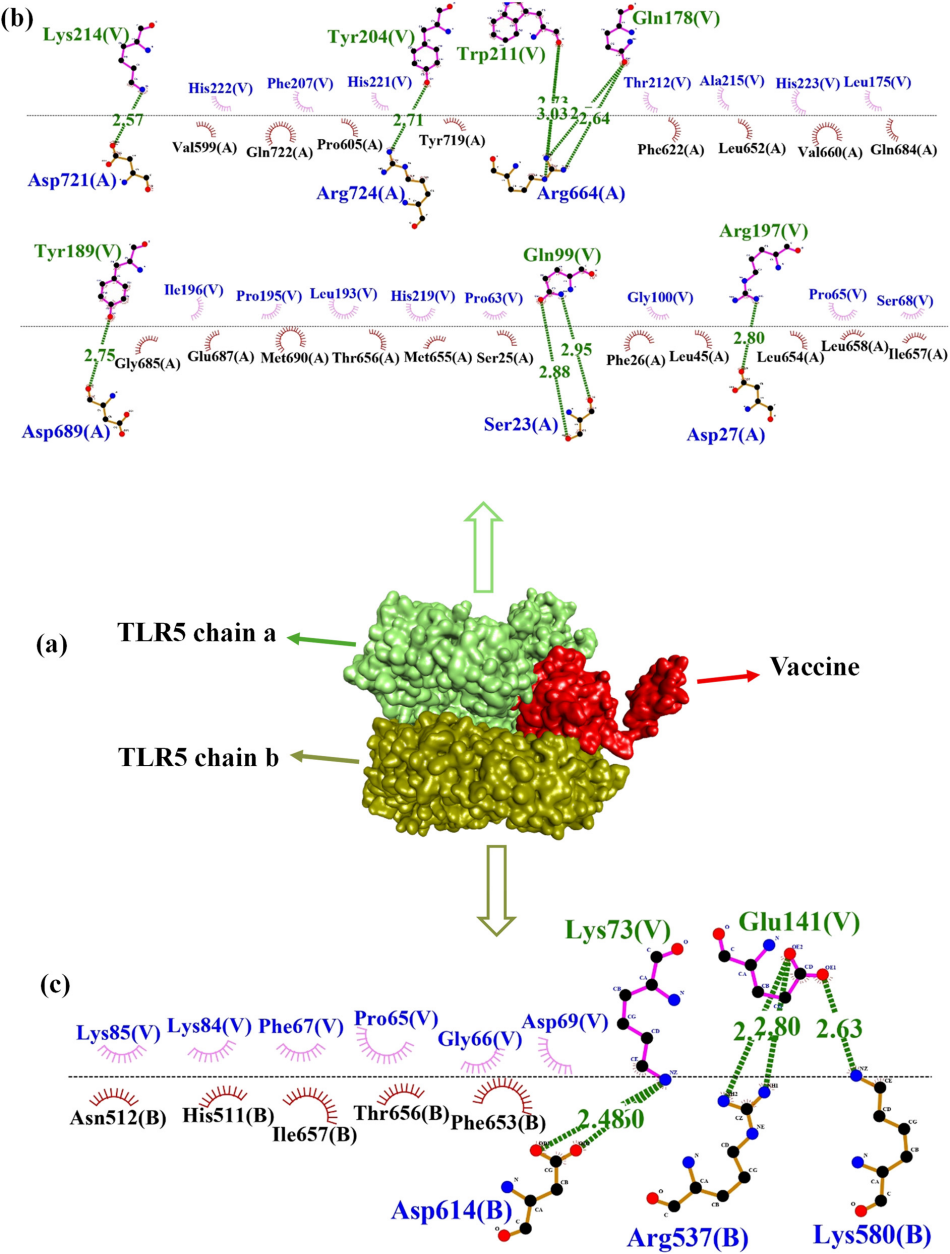
Molecular interaction between the vaccine construct and TLR5 receptor. (a) Docking complex of vaccine with the TLR5. (b) And (c) interaction of vaccine candidate with chain A and chain B of TLR5 receptor.

The molecular docking of the vaccine protein was performed using ClusPro 2.0 with human Toll-like receptors 3 and 5, and validated with the HDOCK server. ClusPro produced a total of 29 distinct models for both TLR3 and TLR5. The TLR3 vaccine candidate showed the lowest energy of −1,008.3 , with 56 members and 13 hydrogen bonds found between the vaccine and the TLR3 complex. However, the vaccine-TLR5 complex showed the lowest energy of −1,309.2, having 75 members and with the presence of 15 hydrogen bonds. The docking outcomes were substantiated through a comprehensive analysis of the linkage between the vaccine and the receptor. The docking results showed that the vaccine-TLR5 complex exhibited an energy of −360.68  with a confidence score of 0.9854, whereas only 4 hydrogen bonds were found between the TLR5 receptor. On the other hand, TLR3 showed a docking score of −335.71  with a binding affinity of 0.9762 to the vaccine, although the energy was slightly lower compared to that of TLR5. TLR3 formed 13 hydrogen bonds with the vaccine, as presented in Table 5. The TLR3 complex formed 13 hydrogen bonds in both ClusPro and HDOCK results with the vaccine. Due to the consistency of TLR3 bond interactions across both tools, HDOCK and Cluspro, TLR3 was selected as the receptor for further analysis. Although TLR5 showed stronger energy scores, the stable and reliable results with TLR3 made it the preferred choice for further molecular dynamics simulation studies. The interactions of docked complexes illustrated in Figure 6 and Figure 7 were examined using LigPlot+v2.2.4.

**Table 5:** Comparative docking results of the vaccine construct with TLR3 and TLR5 receptors.

Complexes	ClusPro 2.0 (lowest energy)	Cluspro members	Hydrogen bonds	Hdock	Confidence score	Hydrogen bonds
Vaccine-TLR3	−1,008.3	56	13	−335.71	0.9762	13
Vaccine TLTR5	−1,309.2	75	15	−360.68	0.9854	4

As visualized by the LigPlot+, 13 hydrogen bonds were found between the vaccine construct indicated with chain A and the TLR3 receptor represented by chain B. Significant interactions include LYS619 with ASP 188 at a distance of 2.5 Å, in addition to TRP 211 interacting with CYS677 at a distance of 2.8 Å, LYS214 with PRO681 exhibiting distances of 2.6 Å respectively. The interacting residues, along with the bond distance of the vaccine and TLR3, are displayed in Table 6.

**Table 6:** Interacting residues of Vaccine with TLR3 receptor.

Sr. No.	Residue name	Residue no.	Chain		Residue name	Residue no.	Chain	Distance (Å)
1	ASP	153	B	–	ARG	12	V	3.8
2	LYS	214	V	–	PRO	681	B	2.6
3	TRP	211	V	–	CYS	677	B	2.8
4	LYS	619	B	–	ASP	188	V	2.5
5	LYS	619	B	–	ASP	188	V	2.5
6	LYS	619	B	–	ALA	185	V	2.7
7	TYR	189	V	–	ASN	596	B	2.8
8	TYR	9	V	–	ASN	180	B	2.7
9	ARG	12	V	–	HIS	156	B	3.0
10	ARG	43	V	–	THR	59	B	2.6
11	LYS	41	B	–	LYS	45	V	2.6
12	LYS	26	V	–	HIS	39	B	2.5
13	ARG	43	V	–	SER	38	B	2.7

The vaccine candidate formed 10 different hydrogen bonds with chain A of the TLR5 receptor, as represented in Figure 7b. The most prominent hydrogen bond was found between LYS214 and ASP721, with a bond distance of 2.5 Å. TYR189 shows interactions with ASP689 at 2.7 Å, ARG724 interacts with TYR204, whereas ARG664 interacts with TRP211, respectively, thereby underscoring significant contact points between the vaccine and the TLR5 receptor chain A, as represented in Table 7.

**Table 7:** Interacting residues of Vaccine with the TLR5 receptor chain A.

Sr. No.	Residue name	Residue no.	Chain		Residue name	Residue no.	Chain	Distance (Å)
1	ARG	724	A	–	TYR	204	V	2.7
2	LYS	214	V	–	ASP	721	A	2.5
3	TYR	189	V	–	ASP	689	A	2.7
4	ARG	664	A	–	TRP	211	V	2.7
5	ARG	664	A	–	GLN	178	V	2.7
6	ARG	664	A	–	GLN	178	V	2.6
7	ARG	664	A	–	TRP	211	V	3.0
8	ARG	197	V	–	ASP	27	A	2.8
9	GLN	99	V	–	SER	23	A	2.9
10	SER	23	A	–	GLN	99	V	2.8

As shown in Figure 7c, the interactions between the vaccine (chain V) and the TLR5 receptor (chain B) displayed five hydrogen bonds. Prominent interactions include LYS 73 of the vaccine establishing a strong hydrogen bond with ASP614 of TLR5 at distances of 2.4 Å and 2.6 Å. Additional CYS 646 interacts with ARG537 to form two hydrogen bonds with GLU141 at distances of 2.7 Å and 2. 8 Å. Furthermore, LYS580 shows interactions with GLU141 at a distance of 2.6  Å as presented in Table 8. These interactions are likely integral to the stability and efficacy of the vaccine’s binding to the receptor, which is essential for activating an immune response.

**Table 8:** Interacting atoms of vaccine with TLR5 receptor chain B.

Sr. No.	Residue name	Residue no.	Chain		Residue name	Residue no.	Chain	Distance (Å)
1	ARG	537	B	–	GLU	141	V	2.7
2	LYS	73	V	–	ASP	614	B	2.6
3	LYS	73	V	–	ASP	614	B	2.4
4	LYS	580	B	–	GLU	141	V	2.6
5	ARG	537	B	–	GLU	141	V	2.8

### Codon optimization and *In silico* cloning

**Figure 8:**
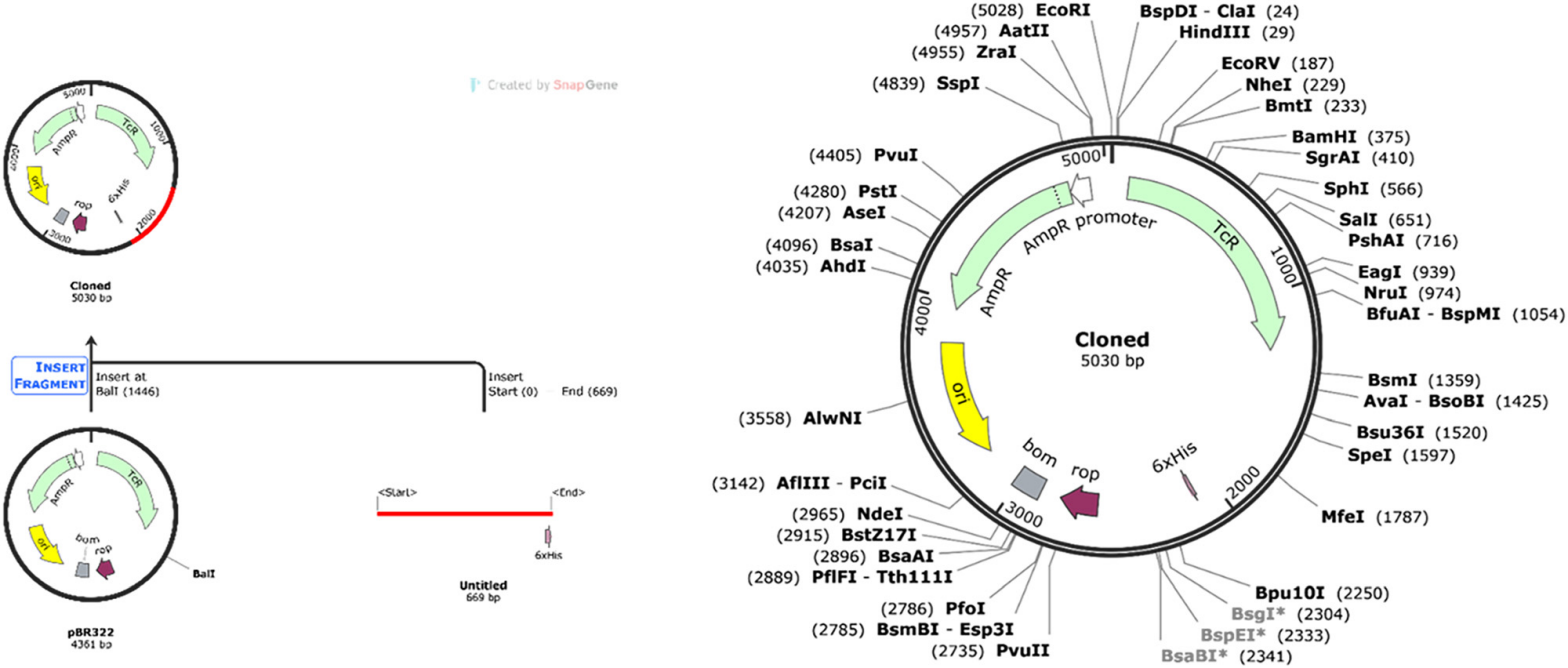
*In silico* cloning of vaccine construct of vaccine in expression vector PBR322.

The vaccine sequence, containing 223 amino acids, was converted into a nucleotide sequence of 669 nucleotide bases. This nucleotide sequence underwent further optimization using the IDT codon optimization tool to enhance vaccine expression in *Escherichia coli*. The vaccine sequence was subsequently converted into a plasmid map using SnapGene software. This map was then incorporated into the vector pBR322 by executing a cleavage at the Bal1 restriction site. The size of the vaccine insert was determined to be 660 base pairs, while the vector size was recorded at 4,361 base pairs. Consequently, in the final cloning system, a cloned vector along with the vaccine candidate map was obtained, yielding an estimated total size of 5,030 base pairs. The cloned vaccine maps is shown in Figure 8.

### Immune simulations

**Figure 9:**
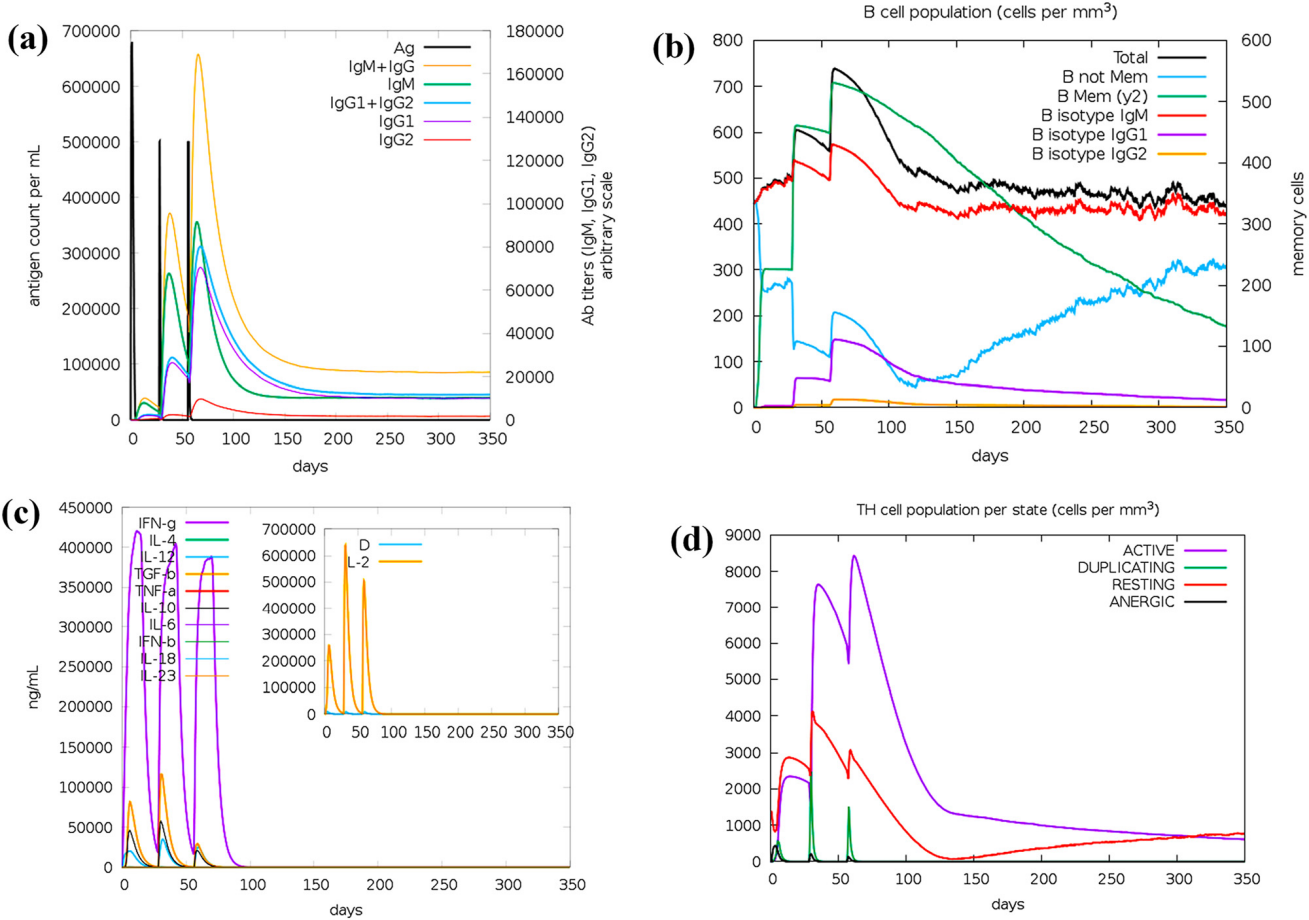
Immune simulation of vaccine (a) representation of antigen and immunoglobulins in immune response. (b) Production of B cells. (c) Cytokines production. (d)T cell population.

C-ImmSim was utilized to evaluate the interaction of the designed vaccine within the host. The results indicated a vigorous immune response characterized by antibody synthesis at the 1, 84, and 180 time steps. After each antigen dose, there was a rapid decline in concentration, with peaks reaching approximately 670,000, 640,000, and 620,000 counts/mL, respectively. A significant increase in antibody levels accompanied this, as the combined IgM and IgG concentration exceeded 120,000 counts/mL following every injection (Figure 9a). Furthermore, evidence of class switching was observed due to the increase in IgG1 and IgG2 levels, which reached 40,000 and 30,000 counts/mL, respectively, with a peak around day 100. These elevated antibody titers, indicating heightened levels, also demonstrated moderate levels (20,000–30,000 counts/mL) throughout the rest of the simulation, signifying enduring humoral immunity and the development of immunological memory (Figure 9b). Following each injection, there was a marked increase in IFN-γ and IL-2, with peaks of approximately 420,000 ng/mL and 65,000 ng/mL, respectively, indicating a strong Th1-type cellular immune response. IL-10 and IL-12 also exhibited moderate transient elevations, which further confirmed the presence of an immunologically balanced profile (Figure 9c). Likewise, helper T cells were highly active, proliferated, and the number of active T helper cells markedly increased after vaccination. The minimal detection of anergic T cells during the entire simulation indicates the absence of immunosuppression or tolerance (Figure 9d). In general, the multiepitope vaccine elicits a robust and well-balanced immune response that integrates both humoral and cell-mediated immunity, conferring sustained protective immunity.

### Molecular dynamics simulations

**Figure 10:**
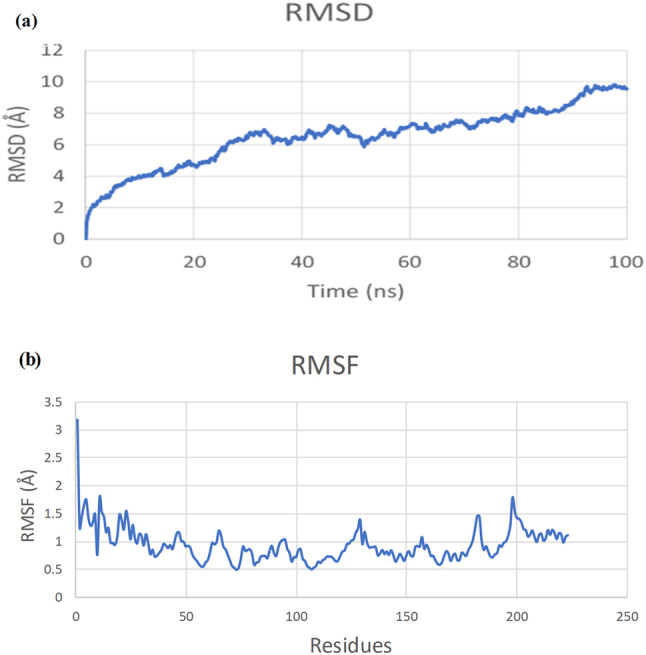
RMSD and RMSF analysis of vaccine-TLR3 complex. (a) RMSD graph of vaccine-TLR3 complex. (b) RMSF graph.

The results obtained from molecular dynamics simulation of the vaccine-TLR3 receptor complex provide a thorough comprehensive of the interaction dynamics, stability, and structural modifications throughout the simulation. These results are based on a range of analytical techniques, including root mean square fluctuation, root mean square deviation, radius of gyration, principal component analysis, and the evaluation of intramolecular hydrogen bonding. The RMSD values exhibit a consistent increase during the early 80–90 ns, reaching approximately 8 Å, indicating that the complex undergoes conformational changes to enhance binding interactions. This phase denotes the conformational rearrangements needed to achieve a stable binding. During the early 25 ns, the RMSD of vaccine-TLR3 complex remained within the range of 2–4.5 Å. However, from 25–85 ns, the complex shows slight fluctuations and RMSD remains constant with 6–8 Å but after the last interval, the RMSD rises to 9.8 Å as shown in Figure 10a. Following this interval, the RMSD curve stabilizes, reflecting the achievement of a stable equilibrium conformation. This behavior highlights the system’s transition flexibility of the vaccine-receptor binding.

The analysis of RMSF indicated minimal fluctuations (2  Å) for the majority of residues, as shown in Figure 10b, thereby indicating strong local stability, alongside minor flexibility observed in particular regions that likely correspond to loop regions or solvent-exposed residues, which are critical for dynamic interactions. These results underscore a strong and stable interaction, with limited flexibility facilitating functional dynamics, thus indicating the vaccine capacity to engage and activate the TLR3 receptor effectively.

**Figure 11:**
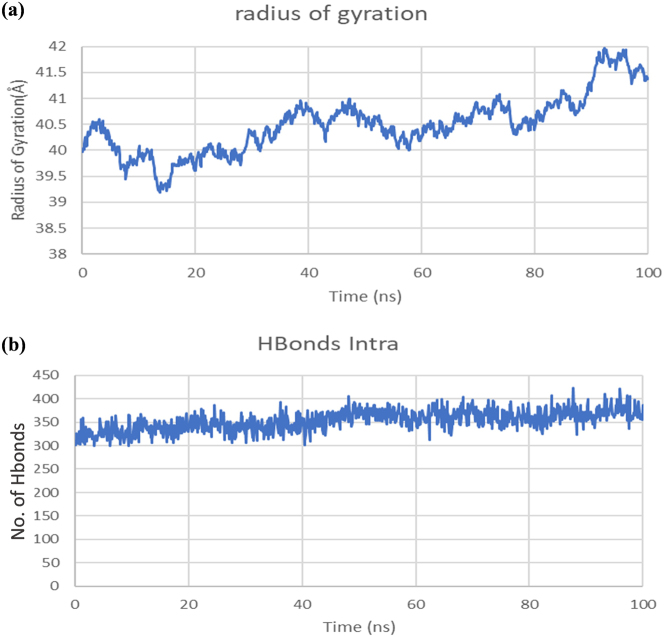
Structural stability analysis of the vaccine–TLR3 complex. (a) Rg graph of vaccine-TLR3 complex. (b) Hydrogen bonds interactions graph between vaccine and TLR3 complex.

Initially, the Rg exhibited fluctuations around 40  Å, with random decreases to 39.5  Å, indicating minor structural adjustments. As the simulation progresses, the Rg revealed a gradual upward trajectory, particularly post 60  ns, ultimately attaining a peak of approximately 41.5  Å by the end of the simulation (Figure 11a). This signifies a slight relaxation or expansion of the complex structure over time, which may reflect adaptive conformational changes within the complex.

The analysis of intramolecular hydrogen bonds yields insights into the stability of internal interactions within the complex. The number of intramolecular hydrogen bonds fluctuated between 300 and 400 throughout the simulation, demonstrating a slight increase during the initial 40 ns, which suggests conformational adjustments. Beyond this interval, the values reach stabilization, reflecting consistent and robust hydrogen bonding interactions. This stability ensures the structural integrity of the complex and its suitability for receptor activation (Figure 11b).

**Figure 12:**
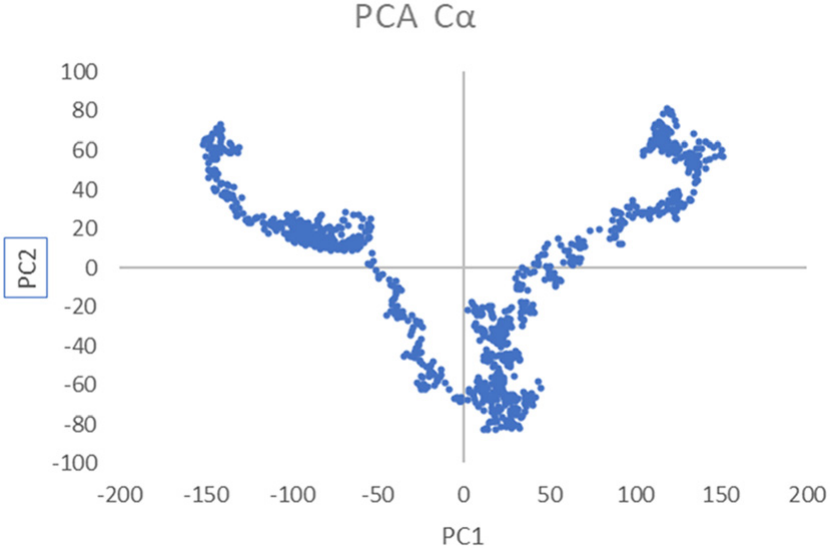
PCA graph of vaccine-TLR3 complex.

Principal component analysis explored the dominant motions and conformational space explored by the complex (Figure 12). The PCA plot exhibited widespread conformational sampling, characterized by significant dispersion along the first two principal components (PC1 and PC2). The ranges of PC1 (−200 to +200) and PC2 (−100 to +100) signify dynamic flexibility, with the trajectory opening distinct conformational states. These transitions exemplify the adaptability of the complex, a crucial feature for effective receptor-vaccine interactions.

## Discussion

Simian Virus 40 is a polyomavirus associated with several human malignancies, including mesothelioma, neural tumors, and non-Hodgkin lymphoma. Regardless of its cancerous capabilities, no vaccine has been developed to prevent SV40 infections [45]. Unlike vaccines that use subunit or live-attenuated viruses, these traditional methods of vaccine development have safety and immunogenicity concerns [46]. However, multiepitope vaccines offer an alternative approach by utilizing immunogenic epitopes from viral proteins to generate strong and long-lasting immune responses [47]. The prediction of B-cell and T-cell epitopes was carried out, resulting in the identification of two important B-cell epitopes and four potential antigenic and nontoxic T-cell epitopes in the two capsid proteins VP1 and VP2 of Simian virus 40. The screened and selected epitopes were incorporated into the vaccine construct using linkers to ensure that the designed vaccine would provide dual immune responses against SV40. Multiepitope vaccine constructs were created by joining B-cell and T-cell epitopes using linkers with the sequences GPGPG, KK, and AAY. The synthesized vaccine demonstrated significant immunogenicity, as indicated by a score of 0.7120, and was classified as non-allergenic, thereby establishing it as a promising vaccine candidate. This designed vaccine construct, composed of 223 amino acids, exhibits an instability index of 26.26, which is generally regarded as a stable and suitable vaccine candidate. The assessment of the physicochemical characteristics of the vaccine further indicated that the engineered construct is thermostable, possesses neutral properties, and exhibits a markedly hydrophilic profile (GRAVY= −0.265).

After tertiary structure prediction and validation of the vaccine protein, molecular docking was performed to assess binding affinity and interactions with immune receptors, including TLR3 and TLR5, yielding scores of −1,008.3  and −1,309.2. The docking results revealed strong interactions with TLR3, facilitated by the presence of 13 strong hydrogen bonds, underscoring the significance of inducing an immune response. This showed that the vaccine binds strongly to immune receptors. These docking results were further cross-validated using the HDOCK server. Although docking results were validated using two independent platforms, ClusPro 2.0 and HDOCK, both of which provide stable interactions, we acknowledge the lack of negative controls in the current study as a limitation. Future research will further incorporate appropriate negative controls, such as scrambled or unrelated peptide sequences, to help ascertain the specificity of the vaccine-receptor interactions. Toll-like receptors are conserved receptors in the body that fulfill an essential function in identifying and mitigating foreign molecules within the host [48]. These receptors play a vital role in identifying antigens linked to pathogens through the mechanism of activation and recognition of conserved regions present in pathogens, such as pathogen-associated molecular patterns (PAMPs) [49].

The vaccine construct underwent immune simulation assessment with three simulated doses administered at intervals of 1, 84, and 180 days over 350 days. The prolonged simulation demonstrated elevated levels of IgG1 and IgG2, active B cells, and sustained cytokine responses (IFN-γ and IL-2), suggesting a long-lasting immunological memory. These results showed potentially prolonged immunity provoked by the vaccine. Nevertheless, due to SV40’s ability for latency and chronic progression, periodic booster doses could enhance immune memory in clinical applications. Experimental validation in animal models will be necessary to determine the precise duration of protection and the optimal timing of boosters. The molecular dynamics simulation results collectively show that the vaccine establishes a stable and biologically significant interaction with the TLR3 receptor. The observed root mean square fluctuation values signify localized stability during 25–85 ns; however, at the end of the simulation, the RMSD increased, showing the flexibility of the complex. This is a limitation of the current study, and it is recommended that future studies increase the simulation time to analyze the stability profile of the vaccine-TLR3 complex. Additionally, the interaction profile of the vaccine can be studied with other immune receptors as well. The root mean square deviation curve validates the system’s progression toward a stable conformational equilibrium. The adaptive structural alterations, as evidenced by the radius of gyration and principal component analysis, further highlight the dynamic flexibility of the complex, which is indispensable for efficient binding and subsequent signaling pathways. The stability of the intramolecular hydrogen bonds strengthens the functional integrity of the vaccine-receptor interaction. These findings strongly validate the vaccine’s potential to activate TLR3, facilitating its practical application in immunological responses.

As there is currently no vaccine available for preventing or treating SV40 infection, this study identifies key epitopes and develops a vaccine construct that may aid in combating SV40 infection. The key benefit of utilizing *in silico* methodologies lies in their capacity to facilitate the swift and effective recognition of virulent genes alongside the development of a multiepitope vaccine [50]. Moreover, *in silico* approaches can offer an extensive degree of precision and specificity in identifying virulent genes, which is imperative for the successful implementation of vaccination strategies [51]. Overall, this study showed successful design and *in silico* validation of a multiepitope vaccine construct against SV40; however, there are still limitations that need to be addressed. This study has limitations, rather than promising results. First, the vaccine construct has only been validated *in silico*, and the immunogenicity and safety profile of the vaccine in humans and animals remains unknown. While immune simulations predicted strong humoral and cellular responses, confirming these results through *in vitro* and *in vivo* experiments is essential. To further evaluate its effectiveness and safety, laboratory studies are needed, including cloning of the vaccine, protein expression in *E. coli*, purification, and testing through immunological assays such as ELISA and cytokine analysis. Furthermore, animal studies will be conducted to assess the immune response, antibody production, and potential side effects. All these studies will complement the computational studies and assess the vaccine’s feasibility for further development.

## Conclusions

Overall, the study highlights the identification of potential T-cell and B-cell epitopes of Simian Virus 40 that can be utilized for vaccine development. The findings underscore the need for a multiepitope vaccine to mitigate the risks posed by SV40, particularly given its ability to infect humans and the potential for transmission through various routes. The proposed vaccine construct, which integrates B-cell and T-cell epitopes alongside adjuvants, demonstrates a comprehensive approach to enhancing immunogenicity and safety. By employing advanced bioinformatics tools for epitope prediction and molecular docking analyses, the study lays a robust foundation for future experimental validation. This research describes a computationally designed multiepitope vaccine with notable immunogenic, structural, and binding characteristics. However, these results require verification through experiments. Subsequent immunological and expression studies through *in vitro* will be conducted, as well as *in vivo* studies, to assess the vaccine’s safety, immunogenic response, and protective efficacy.
